# Advanced Bioinformatics Tools in the Pharmacokinetic Profiles of Natural and Synthetic Compounds with Anti-Diabetic Activity

**DOI:** 10.3390/biom11111692

**Published:** 2021-11-14

**Authors:** Ana Maria Udrea, Gratiela Gradisteanu Pircalabioru, Anca Andreea Boboc, Catalina Mares, Andra Dinache, Maria Mernea, Speranta Avram

**Affiliations:** 1 Laser Department, National Institute for Laser, Plasma and Radiation Physics, 077125 Maurele, Romania; m.a.u.anamaria@gmail.com (A.M.U.); andra.dinache@inflpr.ro (A.D.); 2 Earth, Environmental and Life Sciences Section, Research Institute of the University of Bucharest, University of Bucharest, 1 B. P. Hașdeu St., 50567 Bucharest, Romania; gratiela.gradisteanu@icub.unibuc.ro; 3 “Maria Sklodowska Curie” Emergency Children’s Hospital, 20, Constantin Brancoveanu Bd., 077120 Bucharest, Romania; anca.orzan@gmail.com; 4 Department of Pediatrics 8, “Carol Davila” University of Medicine and Pharmacy, Eroii Sanitari Bd., 020021 Bucharest, Romania; 5 Department of Anatomy, Animal Physiology and Biophysics, Faculty of Biology, University of Bucharest, 91–95 Splaiul Independentei, 050095 Bucharest, Romania; catalina.sogor@bio.unibuc.ro (C.M.); speranta.avram@gmail.com (S.A.)

**Keywords:** diabetes mellitus, natural compounds, QSAR, molecular docking, molecular dynamics, blood–brain barrier, in silico

## Abstract

Diabetes represents a major health problem, involving a severe imbalance of blood sugar levels, which can disturb the nerves, eyes, kidneys, and other organs. Diabes management involves several synthetic drugs focused on improving insulin sensitivity, increasing insulin production, and decreasing blood glucose levels, but with unclear molecular mechanisms and severe side effects. Natural chemicals extracted from several plants such as *Gymnema sylvestre, Momordica charantia* or *Ophiopogon planiscapus Niger* have aroused great interest for their anti-diabetes activity, but also their hypolipidemic and anti-obesity activity. Here, we focused on the anti-diabetic activity of a few natural and synthetic compounds, in correlation with their pharmacokinetic/pharmacodynamic profiles, especially with their blood-brain barrier (BBB) permeability. We reviewed studies that used bioinformatics methods such as predicted BBB, molecular docking, molecular dynamics and quantitative structure-activity relationship (QSAR) to elucidate the proper action mechanisms of antidiabetic compounds. Currently, it is evident that BBB damage plays a significant role in diabetes disorders, but the molecular mechanisms are not clear. Here, we presented the efficacy of natural (gymnemic acids, quercetin, resveratrol) and synthetic (TAK-242, propofol, or APX3330) compounds in reducing diabetes symptoms and improving BBB dysfunctions. Bioinformatics tools can be helpful in the quest for chemical compounds with effective anti-diabetic activity that can enhance the druggability of molecular targets and provide a deeper understanding of diabetes mechanisms.

## 1. Brief Overview of Diabetes Types

Diabetes mellitus (DM) is a metabolic disease defined by a persistently high blood sugar level. There are numerous kinds of diabetes mellitus, but the two most common are type 1 (T1DM) and type 2 (T2DM). T1DM is an autoimmune disease; it occurs due to the destruction of insulin-producing pancreatic β cells, and the patients are entirely reliant on exogenous insulin injection. T2DM is caused by impaired insulin secretion, which generally occurs in the context of pre-existing insulin resistance [1,2]. 

Specific complications may occur faster and progress with early diagnosis and longer exposure to T1DM in children. T2DM is a complex disease dependent on a number of factors such as environmental, metabolic and genetic factors [3]. T2DM affects about 10% of the population, but diagnosing it and maintaining a controlled blood sugar level helps slow down the complications of diabetes [4]. The molecular mechanisms involved in T2DM are incompletely explained, but insulin resistance and defects in insulin secretion are the main causes of this disease [5]. Insulin resistance may be due to both obesity and neuroendocrine function [6].

Macrovascular complications (cerebrovascular, coronary and arterial disease) [7] and microvascular complications (diabetic retinopathy, nephropathy, neuropathy) [8] affect the nervous system, suggesting an inflammatory process that permeates the BBB, thus leading to brain dysfunctions such as those in the psychiatric sphere [9].

With disease progression, chronic exposure to hyperglycaemia induces various types of organ damage [10]. Chronic complications of diabetes are divided into microvascular (i.e., retinopathy, neuropathy and nephropathy) and macrovascular (i.e., cardiovascular disease, mainly as heart failure, coronary heart disease, cerebrovascular and peripheral artery disease). The most accepted mechanism of the deleterious effects of chronic hyperglycaemia is the excessive production of superoxide anion by the mitochondrial electron chain, leading to oxidative stress [11]. Acute complications of T1DM are medical emergencies, consisting mainly of ketoacidosis and hypoglycaemia [12]. Hyperglycaemic hyperosmolar state is a common acute complication in elderly people with T2DM [12]. The main pathogenic event in ketoacidosis is a hormonal disturbance characterized by absolute insulin deficiency in the presence of a relative excess of counterregulatory hormones (i.e., glucagon, catecholamines, cortisol and growth hormone) [12]. 

The BBB is a membrane composed of endothelial cells that protect the central nervous system by preventing the non-selective passage of substances from blood vessels inside the nervous tissue. Diabetes affects the BBB as well, although in a subtle manner, resulting in less data about the subject [13]. Hyperglycaemia may increase the level of pro-inflammatory markers and cell permeability, resulting in decreased efficiency of the BBB [14]. In mice, the hyperglycaemic conditions associated with diabetes lead to a pro-inflammatory phenotype in brain microvessels, with decreased pericyte coverage and increased ICAM-1 expression [15]. The loss of pericytes, doubled by the diminution of tight junctions, results in a permeable BBB [15,16]. 

Herbal medicines are still used in current times and are classified as complementary and alternative medicine. Many plants have anti-diabetic properties via modulating insulin production, cell insulin sensitivity, or glucose absorption. Additionally, to glycaemic management, several plants showed promise in preventing other DM-related illnesses such as cardiovascular problems by lowering cholesterol levels and BMI [17]. Flavanone and polyphenols, natural chemical groups, were investigated as a possible therapy in T2DM or adjuvant in DM treatment. Curcumin, resveratrol, and carotenoid were the most commonly studied substances among these [18].

**Figure 1:**
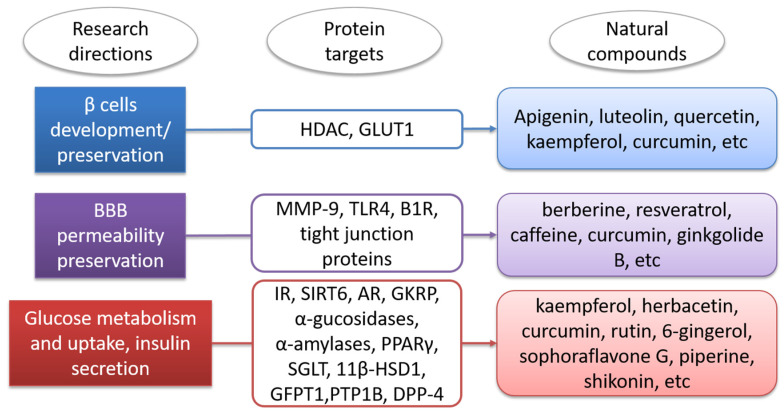
Research directions involving natural compounds for diabetes treatment. The protein targets associated with each direction, as well as some natural compounds that modulate their activities, are presented in the figure. The abbreviations of protein targets are: HDAC—histone deacetylases, GLUT1—glucose transporters 1, MMP-9—matrix metalloproteinases-9, TLR4—Toll-like receptors 4, B1R—bradykinin 1 receptors, IR—insulin receptors, SIRT6—mono-ADP ribosyltransferase-sirtuin-6, AR—aldose reductases, GKRP—glucokinase regulatory proteins, PPARγ—peroxisome proliferator activated receptors gamma, SGLT—glucose co-transporters, 11β-HSD1—11-β hydroxysteroid dehydrogenases type 1, GFPT1—glutamine:fructose-6-phosphate aminotransferases 1, PTP1B—protein-tyrosine phosphatases 1B, DPP-4.

In the present paper, we reviewed studies on natural compounds acting on DM protein targets and on BBB. A schematic representation of our study directions is presented in Figure 1. Initially we discussed the molecular targets in diabetes by considering proteins involved in the metabolism and uptake of glucose, proteins that control insulin secretion and proteins involved in pancreatic β cell development. We identified the natural compounds that could modulate these targets, and we presented bioinformatics studies on these compounds involving the usage of quantitative structure–activity relationships (QSAR), molecular docking and molecular dynamics methods. We also presented databases and web servers useful for the identification of antidiabetic compounds. Concerning the BBB, we identified relevant targets and natural compounds that could prevent BBB dysfunctions. We also presented some platforms useful for calculating the ability of a compound to cross the BBB. 

## 2. Molecular Targets Involved in Diabetes Mellitus

The treatment used in DM aims to prolong life and avoid long-term diabetes-associated complications. Insulin therapy is the primary treatment for T1DM, whereas T2DM is managed with hypoglycaemic medicines, diet, and lifestyle modifications [19]. DM has several receptors that are or may become therapeutic targets; these will be discussed below. 

Insulin receptors (IR) are receptors activated by insulin that have a role in glucose homeostasis. Nowadays, in T1DM, the treatment is represented by self-administrated insulin analogues that activate the IR [20].

Mono-ADP ribosyltransferase-sirtuin-6 (SIRT6) decreased level and function are related to atypical metabolism of glucose and lipids. Mice studies reveal that hypoglycaemia occurs in subjects with SIRT6 deficit [21,22]. Overall, SITR6 has roles in several processes such as regulation of blood glucose, glycolysis, gluconeogenesis, pancreatic β cell function, inflammation, lipid metabolism, etc., and may represent a therapeutic target in DM [23].

Aldose Reductase (AR) is activated in hyperglycaemic conditions and is linked to DM and its complications such as myocardial ischemia, atherothrombotic cardiovascular disease, or diabetes-induced oxidative stress. However, the inhibition of AR may prevent the complications caused by DM [24].

α-glucosidases are enzymes that cleave the oligosaccharides and disaccharides to monosaccharides. Pancreatic α-amylase enzymes catalyse the hydrolysis reaction of α-1,4 glycosidic linkages in many polysaccharides [25]. α-glucosidase and α-amylase inhibitors are used in DM and show better glucose regulation [26,27]. 

Peroxisome proliferator activated receptor gamma (PPARγ) activity can prevent insulin resistance by increasing glucose uptake in adipocyte and muscle cells, which results in lowering of blood glucose levels. Moreover, PPARγ agonists reduce the inflammation mediators that promote insulin resistance and trigger an increase in circulating adiponectin levels with a positive outcome for insulin sensitivity and a decreasing effect on glucose production in the liver [28].

Glucose co-transporter (SGLT) is involved in insulin independent glucose reabsorption in nephrons. SGLT1 and SGLT2 are the main SGLT types, expressed in kidneys in a ratio of 1:10 [29]. The inhibition of SGLTs by gliflozin drugs reduces glucose reabsorption and the levels of glycated haemoglobin [30].

11-β hydroxysteroid dehydrogenase type 1 (11β-HSD1) is an enzyme that converts cortisone to cortisol, which increases hepatic glucose output independent of insulin [31]. 

Glutamine:fructose-6-phosphate aminotransferase 1 (GFPT1) is the rate limiting enzyme in glucose metabolism by the hexosamine pathway (associated with impaired insulin secretion and insulin resistance) [32].

Protein-tyrosine phosphatise 1B (PTP1B) is a protein involved in the insulin signalling pathway and a negative regulator in metabolic disorders [33]. 

Dipeptidyl peptidase-4 (DPP-4) acts on incretin hormones that increase insulin secretion and decrease glucagon secretion [34]. DPP-4 inhibitors have been found in animal research and early clinical trials to considerably decrease fasting and postprandial glucose levels with no risk of hypoglycaemia [35].

Glucokinase regulatory protein (GKRP) represents the endogenous inhibitor of glucokinase, an enzyme that regulates glucose uptake and glycogen synthesis and suppresses glucose production [36]. Glucokinase is involved in glucose homeostasis and is found in pancreatic β-cells and hepatocytes. This kinase stimulates insulin production in pancreatic cells in response to glucose and glucose absorption, glycogen synthesis, and storage in hepatocytes [37]. Hepatic glucokinase expression is reduced in insulin resistance but also T2DM, implying dysregulation of this biomarker [38]. In diet-induced obese mice, the effect of glucokinase activators reduced blood sugar levels [39].

Histone deacetylases (HDAC) modulation appears as an important direction in diabetes therapy, as their inhibition was associated with β cell development, proliferation, differentiation and function [40]. These can be inhibited by several compounds reviewed here [41].

Compared to the other PDKs, pyruvate dehydrogenase kinase 2 (PDK2) has the highest phosphorylation and inactivation of pyruvate dehydrogenase. Since the number of PDK isoforms is increased in diabetes, upregulated PDK2 might be a target for improving glucose tolerance [42]. This kinase is also implicated in hypothalamus inflammation and its consequences (as alteration of feeding behaviour) [43].

GPR40 receptor, also known as free fatty acid receptor 1, is a G-protein-coupled receptor that binds long-chain free fatty acids to improve glucose-dependent insulin production [44]. In a study on diabetic rats, GPR40 activation showed improvements in hyperglycaemia and insulin response [45].

Glucose transporter 2 (GLUT2) may be found in various locations throughout the body, including pancreatic β cells and neurons. GLUT2 is necessary for glucose-stimulated insulin release in pancreatic β cells. GLUT2-dependent glucose-sensing regulates eating, body temperature, and pancreatic β cell mass and function, as well as parasympathetic and sympathetic functions in the central nervous system [46]. Glycogen synthase kinase 3 (GSK-3) is a serine/threonine kinase involved in various processes such as glycogen metabolism, regulation of the cell cycle, and cell proliferation. GSK-3 suppresses the activity of glycogen synthase and insulin receptor substrate-1, two important targets in insulin action. Its enhanced activity under diabetic conditions makes it a promising druggable target in T2DM [47].

Glycerol-3-phosphate dehydrogenase (GPDH) is a mitochondrial enzyme whose inhibition by the antidiabetic drug metformin results in reduced hepatic gluconeogenesis and reduced conversion of lactate and glycerol to glucose [48]. 

## 3. Plants Involved in Diabetes Mellitus Management

At present, several plants have been mentioned in diabetes mellitus management. We present some of them below. 

*Gymnema sylvestre* is a plant rich in phytocompounds that is used as an adjunctive treatment for diabetes and contains, among others, gymnemic acid, gourmarin and gymnapaponins [48]. They have therapeutic effects in diabetes by regulating blood sugar levels. Gymnemic acid is a triterpene saponin with possible antidiabetic action given by the interaction with glyceraldehyde-3-phosphate dehydrogenase (GAPDH) in glycolysis [49]. The extracts from *Gymnema sylvestre* may stimulate insulin secretion and delay glucose absorption from the blood by attaching to intestinal receptors. This way, they decrease the absorption of sugars and limit their passage into the blood [50,51,52]. An interesting study about phytochemicals and the pharmacological and clinical potential of *Gymnema sylvestre* was published by Khan et al. with emphasis on its anti-diabetic activity [53]. The role of some *Gymnema sylvestre* constituents, namely gymnemic acids (I-VII) and gymnemasaponia, in hypoglycaemia has been investigated [51]. These chemicals act by causing the pancreas to secrete more insulin. These compounds are significant because of their structure, which is similar to that of sugar molecules. They attach to taste receptors, blocking the binding site for sugar in food and interfering with the detection of the sweet and bitter taste. These chemicals have a comparable effect on the taste buds and intestines, resulting in a reduction in blood sugar absorption [51]. 

*Momordica charantia* is a plant used in clinical trials that has a beneficial effect on T2DM [54]. Although it had no effect in acute episodes of hyperglycaemia, long-term administration has managed to improve the parameters of patients in clinical trials [55]. The mode of action is not yet fully understood, but studies suggest altered insulin secretion in patients and improved insulin sensitivity by increasing adenosine monophosphate-activated protein kinase (AMPK) [56]. The main chemical compounds found in this medicinal plant are charantine, cucurbitan glycosides, momordicin and oleanolic acids [57,58]. In addition to the presence of natural compounds, *Momordica charantia* can synthesize peptides that can bind to the insulin receptor, lowering blood glucose levels. These peptides may help reduce the need for insulin and limit the side effects of antidiabetic drugs [59].

*Trigonella foenum-graecum* is a medicinal plant whose seeds contain compounds with therapeutic effects. The seeds of this compound can lower the rate of glucose absorption. They help control diabetes, but also reduce cholesterol, cardiovascular risk and other chronic diseases [60].

Ginseng is considered one of the most widely used medicinal plants, with up to thirteen species. Most ginseng-based products come from the *Panax ginseng* and *Panax quinquefolius* species [61]. Therapeutic compounds in ginseng are triterpene glycosides called ginsenosides. Animal studies have shown their involvement in glucose and lipid metabolism and the improvement of biochemical parameters in animal models [62].

Cinnamon can improve blood sugar control and help reduce the complications of diabetes [63]. It is used in traditional Chinese medicine for its various hypoglycaemic, digestive, antispasmodic and antiseptic properties [64].

*Angelica decursive* is a medicinal plant used in traditional medicine in East Asia, with many uses including those as an analgesic, antitussive or tumour suppressor [65,66]. This plant is rich in coumarin compounds, but the most pronounced antidiabetic effect is in the compounds 4′-methoxy Pd-C-I, decursinol, decursidin, 6-carboxylic umbelliferone, 2′-isopropyl psoralen, and Pd-C-III, which has many therapeutic effects. These present inhibitory activity on α-glucosidase and PTP1B [67,68]. PTP1B is a tyrosine phosphatase that regulates the cell cycle and may interfere with the transduction of the insulin stimulus signal [69]. A molecular docking study identified that coumarin binds strongly at the PTP1B site. These results support the importance of these compounds in the prevention and treatment of diabetes [70,71].

*Gynura procumbens* Merr. belongs to the Asteraceae family, and is a plant found in tropical countries that is used for the therapeutic treatment of inflammatory diseases (e.g., rheumatism), heart disease (e.g., hypertension) and diabetic diseases [72]. Studies on the solvent fractions of *G. procumbens* Merr evaluated the antioxidant and antidiabetic effects of the compounds in this plant. In studies on the HepG2 cell line and insulin resistance, *G. procumbens* fractions obtained with the highest phenol content favoured insulin absorption. The compounds with the highest activity in *G. procumbens* were kaempferol, quercitin, caffeoyl-O-hexoside caffeoylquinic acid, coumaroyl-O-hexoside and coumaroylquinic acid. Bioinformatics studies have shown strong molecular interactions between natural compounds and digestive enzymes, thus underlining the value of studying these compounds [73].

*Stachys riederi var.* japonica is a medicinal plant with an antioxidant and antidiabetic effect that can inhibit α-amylase and α-glucosidase. The compounds in this plant have improved glucose uptake into insulin-resistant HepG2 cells. Among the isolated compounds, those with important activity were rosmarinic acid, caffeic acid, oleanolic acid and ursolic acid [74].

*Gardenia jasminoides* extract is used both as a natural dye and as a traditional medicine against various types of diseases such as circulatory diseases. The compounds in this plant are generally bioactive and can have a beneficial effect on the nervous, cardiovascular, and digestive systems, but can also have an anti-diabetic effect [75]. Studies on the methanolic extract from *G. jasminoides* seeds have suggested high antioxidant activity that can inhibit α-amylase and α-glucosidase. The compounds identified with the most promising antidiabetic activity are chlorogenic acid and jasminozide A [76].

*Helianthus tuberosus* is a perennial plant with high resistance to stress, nutritional value and possible antidiabetic effects. This plant is an alternative to classic animal feed; it can produce a high amount of biomass, and its activity on animal digestion, antibacterial, anti-inflammatory and antioxidant effect is due to natural compounds [77].

*Vitex negundo* is a medicinal plant with bioactive properties on glycoprotein metabolism and antihyperglycemic effects due to its ability to suppress the growth of insulin-resistant HepG2 cells. The major compounds are viridiflorol, beta-caryophyllene, sabinen, 4-terpineol, herbacetin rhamnoside, kaempferol, luteolin-7-glucoside, negundoside, p-hydroxybenzoic acid, protocatecuic acid, quinic acid, vitedoin A and vitexin [78]. The functionality of this plant is based on the ability of its active compounds to inhibit α-glucosidase and the uptake of 2,2-diphenyl-1-picryhydrazyl (DPPH) radicals [79].

*Eryngium caeruleum* has antidiabetic and antioxidant potential. Bioactive constituents include thymol, tocopherol, phytol, nerolidol, (I)-neophytadiene, linolenic acid and falcarinol. Molecular modelling studies have shown that *E. caeruleum* interacts with active α-glucosidase sites. Studies in laboratory animals have shown the safety of using *E. caeruleum* extracts, as the chances of them causing adverse reactions are relatively low. The bioactive compounds identified may inhibit α-glucosidase, thus lowering blood glucose [80].

*Curculigo latifolia* has anti-diabetic properties, modulating glucose and lipid metabolism in laboratory rats. The most common compounds in the plant are phloridzine, scandenine, monobenzone, hydroquinone, dimethylcaffeic acid, and hordatin A, compounds with a phytotherapeutic role in the plant that confers anti-diabetic properties. A higher concentration of *C. latifolia* extract increases the percentage of DPP (IV) inhibition [81].

*Limonium axillare* may be an antidiabetic remedy that can reduce hyperglycaemia and restore serum insulin levels by increasing the expression of the glucose transporters GLUT2 and GLUT4. In in vitro studies, *L.* axillare extract had a strong effect of inhibiting α-amylase and ameliorating pancreatic tissue. The inhibition of pancreatic enzymes α-amylase and α-glucosidases may be a mechanism by which the compounds in this plant manifest their antidiabetic capacity. The compounds isolated from *L. axillare* are p-sitosterol-3-palmitate, p-sitosterol, myricetin and gallic acids [82].

## 4. Natural Compounds Involved in Diabetes Mellitus Management

Curcumin is a natural compound found in high amounts in the plant *Curcuma longa* (turmeric) [83]. This compound can relieve symptoms and prolong cell death in T2DM [84]. The effects of this compound have been seen in in vivo, animal and in vitro studies [85]. Curcumin has a molecular mechanism similar to that of thiazolidinedione; an antidiabetic drug that activates the PPAR-γ activated by the peroxisome proliferator [86]. 

Docosanol is a compound that belongs to the class of aliphatic alcohols, with proven antiviral activity [87]. However, molecular docking studies have shown that it is a candidate for inhibiting α-glucosidase and α-amylase [88]. In vitro and in vivo studies show that this compound can lower blood sugar levels [89].

Tetracosanol can also act as an α-glucosidase inhibitor and in combination with a synthetic drug presents increased effectiveness [67]. Anthroquinonol is a derivative of ubiquitin, extracted from *Antrodia cinnamomea* [90]. A study on mice shows that anthraquinone can improve the body’s response to insulin. This compound has an inhibitory effect on dipeptidyl peptidase IV through the kinase cascade activated by adenosine monophosphate [91]. 

Flavones extracted from *Clinacanthus nutans,* and *Nigella sativa* are good candidates as compounds with anti-diabetic activity [92,93]. Rutin is another flavonoid found in many herbs, vegetables and dietary supplements and has an antihyperglycemic effect [94,95]. Its mode of action is not fully understood, but this compound may protect pancreatic cells against apoptosis by decreasing carbohydrate absorption and stimulating insulin secretion [96].

Berberine is a natural alkaloid used to treat fungal and parasitic infections [97]. In diabetes, it has shown its effectiveness by regulating lipid metabolism and improving glycaemic parameters [98,99]. Long-term treatment with berberine can improve insulin secretion. Nevertheless, the mechanism of action is not clear; berberine stimulates glycolysis but can also act as an α-glucosidase inhibitor [99]. Catechin has anti-inflammatory, antidiabetic, and neuroprotective activity [100]. 

Herbacetin has a favourable effect in maintaining blood sugar levels at normal levels. If intervenes in gluconeogenesis, thus mediating the metabolic pathway and preventing the overproduction of glucose [101]. The compound targets liver fructose 1,6-biophosphatase, and thus studies have shown that it may be a valid alternative in the treatment of patients [71,102]. 

Kaempferol is a natural polyphenol studied for its antidiabetic role. Kaempferol treatment ameliorated histological changes in diabetes-induced renal tissue by inhibiting Rho-kinase [103,104]. Molecular docking studies have shown that kaempferol targets α-glucosidase with high-affinity binding, resulting in an inhibitory effect [105] Molecular docking scores and studies in mice have shown that Leucodelphinidin has an antidiabetic effect [106]. 

Isorutarine is linked to the main target of antidiabetic drugs, α-glucosidase and α-amylase. The same targets are inhibited by actinodafine, a compound with antidiabetic activity [88]. The proposed molecular mechanism for this compound came from molecular docking studies, and its effectiveness has been proven by studies in laboratory animals. Additionally, this compound has high therapeutic potential in lowering blood sugar levels [67]. Nodakenin has an inhibitory effect on α-glucosidase, PTP1B, acetylcholinesterase and butyrylcholinesterase [107].

In in vitro studies, compounds such as neochlorogenic acid, chlorogenic acid, caffeic acid, 5-OA-(4-cumaroyl) -quinic acid, feruloylquinic acid, caffeoylquinic acid, isoxazolidine, and β-D-glucoside of salicylic acid showed antidiabetic activity, acting on α-amylase and α-glucosidase. Free radical scavenging and inhibition of diabetes-associated enzymes are dose-dependent, but according to a study by Mariadoss et al., phytocompounds could reduce blood sugar levels, triggering glucose uptake into insulin-resistant HepG2 cells [108]. 

Molecular docking studies on (4Z, 12Z)-cyclopentadeca-4, 12-dienone have shown that this compound can inhibit the action of enzymes aldose reductase, glucokinase, pyruvate dehydrogenase kinase, receptor-gamma, glycogen synthase kinase-3, and fructose-6-phosphate amidotransferase with a role in diabetes. This compound is a valid candidate for the development of new antidiabetic drugs due to the various molecular targets to which it may bind [109].

## 5. Quantitative Structure–Activity Relationships (QSAR) Predicted Anti-Diabetic Activity 

Quantitative structure–activity relationships (QSAR) investigations are a fast computational approach for predicting a compound’s biological activity from its chemical structure. QSAR techniques aid medicinal chemists in comprehending the link between hypoglycaemic action and molecular characteristics [110,111,112]. Statistical measures such as the cross-validated correlation coefficient, fitted correlation coefficient, and standard deviation of error prediction are commonly used to assess QSAR investigations [113]. The dimensions of QSAR models range from 0 to 6; however, the most common are 2D QSAR and 3D QSAR. Geometric characteristics, topological indices, and molecular fingerprints are all considered for 2D QSAR models, but steric properties are not. The spatial characteristics of the compound are the focus of the 3D QSAR method [114]. 

In silico methods were applied by Gajjar et al. to a series of 3-aryl-3-ethoxypropanoic acid derivatives as modulators of GPR40. Two 3D-QSAR models (utilizing CoMFA and CoMSIA), and a 2D-QSAR model (using HQSAR technique), were used to determine the connection between the structure and biological activity of these compounds. All QSAR models have good statistical parameters [44]. 

The QSAR models analysed contour maps of lipophilic, electrostatic, hydrophobic, donor, positive and negative contributions. The findings revealed that 3-aryl-3-ethoxypropanoic acid derivatives might be effective anti-diabetic medicines that target the human GPR40 receptor [44]. Izadpanah et al. [115] conducted QSAR and molecular docking analyses on a set of 35 α-glucosidase inhibiting compounds. Statistical parameters in the research suggested a successful prediction model. The most active compound on α-glucosidase showed high inhibitory activity of 9.22, which implies that it might be used to treat T2DM [115].

Flavone compounds exhibited a potential inhibitory effect on the a-glucosidase enzyme in a QSAR investigation. The results of 20 flavone derivatives (1–20) were compared to the α-glucosidase inhibitor acarbose in this study. The IC50 values of the flavone derivatives varied from 1.02 to 38.1 M, according to the findings. These results revealed that acarbose (IC50 = 39.45 0.11 M) had lower inhibitory activity [116]. Maurya et al. used a 3D-QSAR model to identify positions and types of groups that increased the activity of 116 coumarin derivatives against lysosomal α-glucosidase. The study showed that the binding affinity of lysosomal α-glucosidase antagonists can be improved by replacing H-bond donor groups on the coumarin ring moieties at the C3, C5, and C7 positions, respectively. Additionally, binding an H-bond donor to the attached carbon rings and oxygen atoms can improve the compound’s activity [117].

Xu et al. built 2D QSAR models to characterize the important fragments of a series of 25 andrographolide derivatives. In addition, 3D QSAR models were created to investigate the spatial distribution of their main groups. To efficiently detect the fragments and their spatial distribution, they merged the 2D and 3D QSAR models. Derivatives 20–23 of the 25 andrographolide compounds had a strong inhibitory effect on the α-glucosidase receptor, while compounds 3, 4, 13, and 16 had low inhibitory activity [118].

Ghamali et al. used 44 substituted flavonoids with previously experimentally determined inhibitory activity on AR to develop three QSAR models (a multiple regression analysis, a nonlinear regression, and an artificial neural network model). The models had high stability and prediction power for flavonoid derivative inhibitory action against AR. The artificial neural network model is the best QSAR model, and it may be useful to predict the inhibitory effects of flavonoid derivatives [119]. 

## 6. Molecular Docking and Molecular Dynamics Predicted Anti-Diabetic Activity

Molecular docking simulations usually predict the interaction between a compound and a specific target protein [120,121]. Flavonoids or other chemical classes of compounds from traditional medicinal plants are frequently used in molecular docking studies to find the specific compounds responsible for the positive effect [122]. 

Here, we will present several studies that use the molecular docking approach to predict the binding affinity of compounds from plants that interact with DM-specific targets. In the case of some promising compounds, the stability of receptor-ligand complexes was addressed by molecular dynamics (MD) simulations.

Molecular docking studies show that compounds from plants such as *Ficus benghelensis*, *F. racemosa*, *F. religiosa*, *Thespesia populena*, and more have the potential to bind to targeted receptors in DM [67,71,117] (Table 1). For both α-amylase and α-glucosidase targets, curcumin presents the lowest dock-score (Table 1) [67]. A chemical with a low free binding energy has a better chance of binding to that target. The lower the binding energy, the greater the chance of binding [123,124]. These simulations from Jhong’s study were experimentally validated (in the same study) and compared with acarbose (α-amylase inhibitor used in the treatment of DM). Jhong’s study concluded that curcumin and actinodaphnine, interacting with α-glucosidase, and curcumin and berberine, interacting with the α-amylase, present a higher IC50 activity compared with acarbose [67]. 

Another docking study on α-glucosidase conducted by Maurya concluded that isorutarine, a coumarin analogue, has a good docking score (Table 1) [117]. Singh’s study used as DM targets the IR, AR and SIRT6 receptors. It showed that kaempferol had the lowest binding energy (kcal/mol) in interaction with AR, gossypetin in interaction with IR, and sorbifolin in interaction with the SIRT6 receptor (Table 1) [71]. Sathiyaseelan et al. evaluated the antidiabetic effect of phytochemicals from *Gynura procumbens* methanolic extract and its various solvent fractions on α-amylase and α-glucosidase receptors. They also predicted the interaction of the main compounds identified in *G. procumbens* extract with porcine pancreatic α-amylase and α-glucosidase, using a molecular docking approach (Table 1) [73].

*Stachys riederi* var. *japonica* solvent extract and fractions were analysed on induced T2DM mice. Saravanakumar et al. study also predicted the binding affinity of identified compounds from *Stachys riederi* var. *japonica* for α-amylase and α -glucosidase receptors using molecular docking (Table 1) [74]. Anti-diabetic effects of compounds identified from *Gardenia jaminoides* and *Helianthus tuberosus,* were also predicted using molecular docking (Table 1) [76,108]. 

The anti-diabetic activity of (4Z, 12Z)-cyclopentadeca-4, 12-dienone from the Grewia hirsute plant was investigated by Natarajan et al. in a molecular docking study. They tested the anti-diabetic efficacy of the compound on seven molecular targets, such as AR or GFPT1 (Table 1) [109]. *Vitex negundo* leaf constituents were identified through ultra-high-performance liquid chromatography-quadrupole time of flight/tandem mass spectrometry, and the anti-diabetic properties were evaluated on α-glucosidase using molecular docking models (Table 1) [79].

The hypoglycaemic activity of insulin-like peptides from *Momordica charantia* was determined using a molecular docking approach. The study investigated the activity of several peptides such as LIVA, EKAI, EALF, DFGAS and EPGGGG on four target proteins, namely the experimentally determined structures of IR, SGLT1, dipeptidyl peptidase-IV, and the predicted 3D structure of GLUT2 (Table 1) [59]. In the Sadiq et al. study, bioactive components in *Eryngium caeruleum* were discovered using GC-MS and HPLC-DAD investigations. Several molecular docking models were used to determine the binding energy of the reported molecule and compared with acarbose. All substances could inhibit the α -glucosidase receptor, according to the investigations (Table 1) [80].

Zabidi et al. identified the main compounds from *Curculigo latifolia* and, using molecular docking analyses, they predicted the binding affinity of those compounds for α-glucosidase, DPP-4 and IR (Table 1). They compared the results with the reference drugs for each target acarbose (−7.4 kcal/mol, on α-glucosidase), sitagtiptin (−8.8 kcal/mol on DPP-4), and insulin (−7.9 kcal/mol on IR) [81].

According to the observations of Abdel-Sattar et al., the root extract of *Limonium axillare* exhibits anti-diabetic properties such as raising insulin secretion, increasing GLUT2 and GLUT4 expression, and thereby increasing glucose absorption. The root extract’s main ingredients may have a binding affinity for GPDH, according to molecular docking analyses (Table 1) [82].

**Table 1:** Target receptors, the natural compounds with the best docking results from each study, docking scores and software used for prediction.

**Target**	**Compounds**	**Predicted Energy of Binding (kcal/mol)**	**Software Used**	**References**
AR (PDB: ID:1US0 [15]) Organism: Homo sapiens	kaempferol	−10.034	YASARA [125]	[71]
	herbacetin	−9.623		
	sorbifolin	−9.391		
IR (PDB: ID:1IR3 [16]) Organism: Homo sapiens	gossypetin	−8.429	YASARA [125]	[71]
	herbacetin	−8.165		
	sorbifolin	−8.063		
SIRT6 (PDB ID: 3K35 [17]) Organism: Homo sapiens	gossypetin	−8.569	YASARA [125]	[71]
	herbacetin	−8.632		
	kaempferol	−8.533		
	sorbifolin	−8.697		
**Target**	**Compound**	**Dock** **Score (-Potential of Mean Force)**	**Software Used**	**References**
α-glucosidase (PDB 2ZE0 [126]) Organism: Geobacillus sp. HTA-462	curcumin	−153	LigandFit implemented in DS 2.5 (DS, Accelrys Software, San Diego, CA, USA)	[67]
	antroquinonol	−180		
	rutin	−159		
α-amylase (PDB 1HNY [127]) Organism: Homo sapiens	curcumin	−175	LigandFit implemented in DS 2.5 (DS, Accelrys Software, San Diego, CA, USA)	[67]
	16-hydroxy-cleroda-3,13-dine-16,15-olide	−155		
	docosanol	−154		
	berberine	−142		
	catechin	−135		
	quercetin	−132		
	rutin	−126		
**Target**	**Compound**	**Docking Score (kcal/mol)**	**Software Used**	**References**
Lysosomal α-glucosidase (PDB ID: 5KZX [128]) Organism: Homo sapiens	Isorutarine	−7.64	Maestro 12.0 of Schrödinger LCC, New York, NY, USA	[117]
	2′Isopropylpsoralene	−6.64		
	4-hydroxy d-C-III	−6.45		
**Target**	**Compound**	**Predicted Energy of Binding (kcal/mol)**	**Software Used**	**References**
porcine pancreatic α-amylase (PDB ID: 1OSE [129]) Organism: Sus scrofa	Caffeoylquinic acid	−10.33	Argus lab 4.0.1 [130]	[73]
	O-Coumaroylquinic acid	−10.01		
	Coumaroyl-Ohexoside	−9.75		
α-glucosidase (PDB ID: 3A4A [131]) Organism: Saccharomyces cerevisiae	Caffeoylquinic acid	−10.84	Argus lab 4.0.1 [130]	
	O-Coumaroylquinic acid	−10.65		
	Coumaroyl-Ohexoside	−10.60		
**Target**	**Compound**	**Binding Affinity** **(kcal/mol)**	**Software Used**	**References**
human pancreatic α-amylase (PDB ID: 5E0F [132]) Organism: Homo sapiens	Ursolic acid	−9.8	Autodock Vina 1.1.2 [133]	[74]
	Oleanolic acid	−8.7		
	Rosmarinic acid	−8.5		
human lysosomal acid α-glucosidase (PDB: 5NN8 [134]) Organism: Homo sapiens	Ursolic acid	−8.2		
	Oleanolic acid	−8.2		
	Rosmarinic acid	−8.2		
human pancreatic α-amylase (PDB: 5E0F [132]) Organism: Homo sapiens	Chlorogenic acid	−8.7	Autodock Vina 1.1.2. [133]	[76]
	Jasminoside A	−8.7		
	Jasminoside F	−8.5		
human lysosomal acid α-glucosidase (PDB: 5NN8 [134]) Organism: Homo sapiens	Acarbose derived trisaccharide	−8.7		
	Acarbose	−8.7		
	Chlorogenic acid	−8.2		
**Target**	**Compound**	**Predicted Energy of Binding (kcal/mol)**	**Software Used**	**References**
porcine pancreatic α-amylase (PDB ID: 1OSE [129]) Organism: Sus scrofa	cryptochlorogenic acid	−9.860	ArgusLab 4.0.1 [130]	[108]
	feruloylquinic acid	−8.613		
	neochlorogenic acid	−7.452		
α-glucosidase (PDB ID: 3A4A [131]) Organism: Saccharomyces cerevisiae	caffeoylquinic acid	−10.737		
	neochlorogenic acid	−10.732		
	cryptochlorogenic acid	−10.632		
**Target**	**Compound**	**Docking Score**	**Software Used**	**References**
AR (PDB ID: 3G5E [135]) Organism: Homo sapiens	(4Z,12Z)-cyclopentadeca-4, 12-dienone	−7.61	GLIDE 5.0 of Schrödinger LCC, New York, NY, USA [136]	[109]
glucokinase (PDB ID: 4IXC [137]) Organism: Homo sapiens		−6.18		
PDK2 (PDB ID: 4MP2 [138]) Organism: Homo sapiens		−5.21		
PPARγ (PDB ID: 3DZY [139]) Organism: Homo sapiens		−7.57		
GSK-3 (PDB ID: 3F7Z [140]) Organism: Homo sapiens		−6.01		
11β-HSD1 (PDB ID: 4K1L [141]) Organism: Homo sapiens		−7.85		
GFPT1 (PDB ID: 2ZJ4 [142]) Organism: Homo sapiens		−5.57		
**Target**	**Compound**	**Docking Score (kcal/mol)**	**Software Used**	**References**
α-glucosidase (predicted 3D structure) Organism: Saccharomyces cerevisiae	casticin	−8.452	MOE, Chemical Computing Group, Monreal, Canada	[79]
	negundoside	−7.923		
	herbacetin rhamnoside	−7.369		
**Target**	**Compound**	**S-Score**	**Software Used**	**References**
IR (PDB: ID:1IR3 [16]) Organism: Homo sapiens	KDDGHL	−18.56	MOE, Chemical Computing Group, Monreal, Canada	[59]
	EPGGGG	−16.71		
	TSEP	−15.66		
SGLT1 (PDB ID: 3DH4 [143]) Organism: Vibrio parahaemolyticus	ESIRD	−23.81		
	DSRHR	−23.64		
	RRKKV	−20.64		
dipeptidyl peptidase-IV (DPP (IV))(PDB ID: 4A5S [144]) Organism: Homo sapiens	PTRHM	−10.1067		
	RRKKV	−9.9189		
	KDDGHL	−9.4991		
GLUT2 (predicted 3D structure)	RRKKV	−10.5970		
	RSIHEP	−10.5171		
	ERFDSG	−9.6986		
**Target**	**Compound**	**Binding Energy**	**Software Used**	**References**
α-glucosidase (predicted 3D structure)	tocopherol	−7.7008	MOE, Chemical Computing Group, Monreal, Canada	[80]
	linoleic acid	−7.1746		
	phytol	−7.0629		
**Target**	**Compound**	**Binding Affinity** **(kcal/mol)**	**Software Used**	**References**
α-glucosidase (PDB ID: 4J5T [145]) Organism: Saccharomyces cerevisiae S288C	phlorizin	−8.2	AutoDock [133]	[81]
	scandenin	−8.0		
	pomiferin	−8.0		
DPP-4 (PDB ID: 2P8S [146]) Organism: Homo sapiens	phlorizin	−10.9		
	pomiferin	−9.6		
	mundulone and scandenin	−9.3		
IR (PDB: ID:1IR3 [16]) Organism: Homo sapiens	phlorizin	−7.0		
	mundulone	−6.9		
	pomiferin	−6.6		
**Target**	**Compound**	**Docking Score (kcal/mol)**	**Software Used**	**References**
GPDH (PDB ID: 1WPQ [147]) Organism: Homo sapiens	2′,4′ dihydroxychalcone	−6.2652	MOE, Chemical Computing Group, Monreal, Canada	[82]
	compound 4	−5.7992		
	compound 3	−5.6075		

Arif et al. [59] also evaluated the complex binding energies of peptides to selected targets by applying the molecular mechanics generalized born surface area (MM-GBSA) method on 50 ns trajectories obtained by MD simulations of LIVA-IR and DFGAS-SGLT1 complexes. MD simulations revealed the stability of complexes, and calculated binding energies showed significantly favourable interactions between ligands and targets. The ligands appear to be stabilized at the binding sites by van der Waals energy, the nonpolar energy term being the most important for complex formation [59].

An extensive screening study performed on a library of 257 compounds from medicinal plants with antidiabetic activity identified 79 potential inhibitors of α-amylase [148]. Six phytochemicals (shahidine, epicatechin, quercetin, isocolumbin, ellagic acid, lutolin) were selected by re-scoring, ADMET and drug-likeness analysis. MD simulations (30 ns long simulations) confirmed the stability of complexes formed by α-amylase (PDB ID: 3BAJ [149]) and these compounds, supporting their potential to inhibit the enzyme [148]. 

The inhibition of α-glucosidase by natural compounds from spices such as fenugreek, black pepper, ginger and turmeric was investigated in conjunction with their agonistic activity on PPARγ [150]. Curcumin, pipernonaline, 6-gingerol and trigonelline were docked at α-glucosidase (PDB ID: 5NN8 [134]) and PPARγ (PDB ID: 4A4V [151]) binding sites, and replica exchange MD simulations were performed to characterize the dynamical behaviour of complexes [150]. Results showed that curcumin and pipernonaline formed stable complexes with the two proteins, which supports the beneficial effects of the compounds in diabetes [150]. 

Salaudden et al. [152] tested 10 natural compounds with proved antidiabetic activity against SGLT1 and SGLT2 using molecular docking and filtered them by ADMET, drug-likeness and lead-likeness analysis. The most promising compound was sophoraflavone G, which was also proved to form stable complexes with SGLT2 using MD simulations [152]. Since the crystal structures of SGLT1 and 2 are unknown, the authors performed all calculations using homology models [152]. 

Other DM targets and their modulation by natural compounds from *Piper longum* Linn. were addressed by Thakurla et al. [153]. The targets they investigated were: 11β-HSD1 (PDB ID: 1XU7 [154]); GFPT1 (PDB ID: 2V4M [155]); PTP1B (PDB ID: 3SME [156]); DPP-4 (PDB ID: 1J2E [157]); and GKRP (PDB ID: 4BBA [158]. The bioactive compounds that were analysed were retrofractamide A, piperine, piperlongumine, and piperlonguminine, all drug-like compounds. The molecular docking of compounds at selected proteins led to good results, with piperine being the most promising compound [153]. 

Vo et al. [159] investigated the modulation of 11β-HSD1, GFPT1, PTP1B and SIRT6 by 20 bioactive compounds from *Euphorbia thymifolia* Linn. Molecular docking of compounds at their possible targets allowed the identification of seven compounds able to bind all targets, namely: β-amyrine, teraxerol, 1-O-galloyl-β-D-glucose, corilagin, cosmosin, quercetin-3-galactoside and quercetin [159]. 

The ability of shikonin, a natural naphthoquinone dying pigment, to inhibit PTP1B was evaluated through a complex approach involving in silico and in vitro methods [160]. Shikonin was docked at its putative binding site from 1AAX structure [161], which allowed the identification of crucial residues involved in the interaction. The ZINC Natural Product database was interrogated for compounds with pharmacophore features similar to those of shikonin, resulting in a library of 1860 compounds that were screened against PTP1B structure. Shikonin and the 100 best docked compounds were filtered based on their ADMET and drug-likeness properties, which led to the identification of four additional possible ligands: ZINC31168041, ZINC31168045, ZINC31168041 and ZINC31168554. These compounds were also docked at PTP1B active sites, and ZINC31168045 presented the highest docking score [160]. MD simulations also suggest that shikonin could be a lead molecule for inhibiting PTP1B. The powerful inhibition of PTP1B by shikonin was determined experimentally (IC50 = 8.72 M), confirming the antidiabetic effects of the shikonin scaffold [160]. 

Flavones are good candidates to inhibit HDAC1 and HDAC2. The docking of vorinostat (a known inhibitor of the two enzymes), flavone, apigenin and luteolin to HDAC1 and HDAC2 structures was performed at a vorinostat binding site revealed by X-ray crystallography (PDB ID: 4LXZ [162]) [163]. Their results show that flavone, apigenin and luteolin can occupy vorinostat binding sites and can interact with enzymes with energies similar to vorinostat. Such data support the idea that dietary flavones can be used for epigenetic therapy [163].

## 7. Anti-Diabetic Synthetic Compounds and Their Molecular Target Effects on BBB

DM is a disorder that can lead to BBB disruption and cognitive decline. Ischemic stroke, atherosclerosis, vascular cardiac consequences, hemodynamic abnormalities, cognitive deficits, neurochemical, electrophysiological and behavioural alterations are all linked to hyperglycaemia and insulin resistance [164,165]. BBB disruption and inflammation are significant elements in diabetic stroke that lead to a poor outcome. Microcapillary integrity and oxidative stress may play a role in the overexpression and activation of the receptor for advanced glycation end products (RAGE). T2DM and Alzheimer’s disease (or “type 3 diabetes”) are linked because type I membrane protein carries amyloid-beta across the BBB [165]. Matrix metalloproteinases (MMP) are known to exacerbate white matter damage and are linked to BBB disruption [166]. During induced epileptic episodes, the permeability of the BBB is affected in T1DM. BBB permeability increased significantly in seizures under diabetic circumstances, and BBB damage increased during epileptic seizures [167].

**Figure 2:**
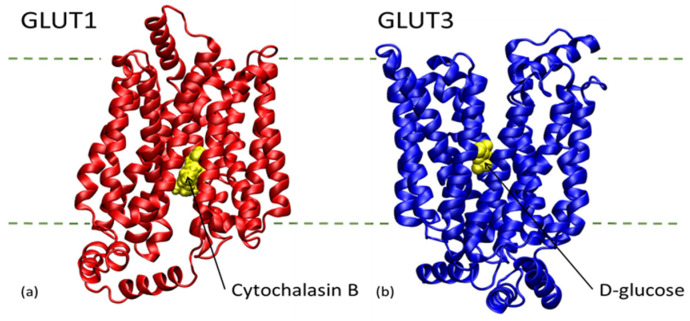
(**a**) Structure of GLUT1 in complex with cytochalasin B, according to the crystal structure 5EQI [173]. (**b**) Structure of GLUT3 in complex with D-glucose according to the crystal structure 4ZW9 [176]. The ligands cytochalasin B (**a**) and D-glucose (**b**) are represented with yellow van der Waals spheres.

Glucose transporter 1 (GLUT1) and glucose transporter 3 (GLUT3) are the most important glucose transporters across the BBB [168]. According to Prasad et al., the mRNA and protein expression of GLUT1 and GLUT3 are down-regulated in hyperglycaemia and increased in hypoglycaemia [165]. According to Duelli and Kuschinsky, the GLUT1 receptor level decreased by 8% after 3 weeks of hyperglycaemia, while GLUT3 transporter levels stayed constant [168]. GLUTs 1, 3, and 4 were dramatically reduced in the brains of untreated diabetic mice by 61, 69, and 64%, respectively [169]. The blockage of GLUT1 expressed in autoreactive T cells could limit the destruction of pancreatic β cells in T1DM. As promising as such a pharmaceutical approach could be, it could interfere with GLUT1 activity in BBB, leading to neurological symptoms. An effective therapeutic approach against T1DM autoimmunity with less off-target side effects should be limited in time, taking into account the age of patients and the characteristics of their T cell response [170]. Several GLUT1 inhibitors have been developed over the years for cancer treatment [171]. Cytochalasin B is a mycotoxin that blocks GLUT1 [172]. By solving the crystal structure of GLUT1 in complex to cytochalasin B, Kapoor at al. [173] described the interactions between the ligand and the receptor, opening the possibility to develop even more specific and effective inhibitors. The structure of the complex is presented in Figure 2a. 

GLUT3 is similar in structure to GLUT1, but with different physiological roles and transport affinity [174]. GLUT3 was associated with cell invasion and cancer metastasis, being an attractive anti-cancer drug target, especially in brain cancers such as glioblastoma [175]. The structure of GLUT3 with a D-glucose molecule bound in its binding site is presented in Figure 2b.

The matrix metalloproteinase enzymes may have a role in BBB breakdown due to their systemic activation during diabetic ketoacidosis [177]. The findings of Hoffman et al. [177] indicated that the matrix metalloproteinase 9 (MMP-9) is expressed in the fatal brain oedema of diabetic ketoacidosis patients as well as on cells from brain intravascular regions. MMP-9 is present on neurons in the hippocampus areas of both brain oedema and diabetic ketoacidosis patients. At the same time, the Tissue Inhibitor of Metalloproteinases 1 (TIMP1) expression in the locations is reduced [177]. The authors suggested that further studies are necessary to determine the role of MMP-9 in the pathogenesis of the neurologic catastrophe of brain oedema in diabetic ketoacidosis. Inhibition of MMP-9 expression might help preserve neuronal function and BBB integrity during diabetic ketoacidosis [177]. 

**Figure 3:**
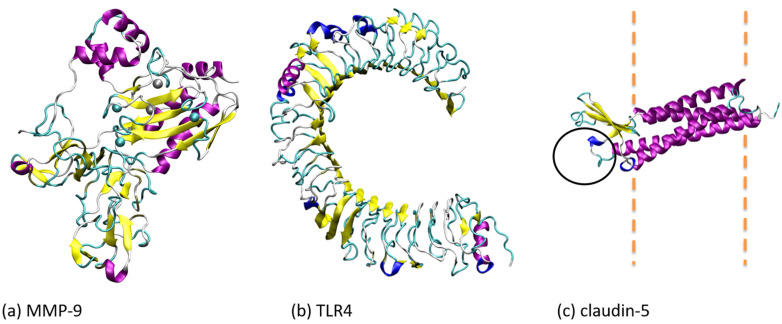
Three-dimensional structures of some proteins associated with BBB breakdown, namely matrix metalloproteinase-9 (MMP-9) and Toll-like receptor 4 (TLR4), or with BBB proper function, namely claudin-5. In (**a**) we represented MMP9 according to the 3D structure 1L6J [181]. In (**b**) we represented TLR4 according to 3FXI structure [182], and in (**c**) we represented the structural model of claudin-5 generated using the machine learning approach AlphaFold [183] that we retrieved from AlphaFold Protein Structure Database [184]. In the case of claudin-5, we used a black circle to show the location of a permeation pore defined by two claudin-5 dimers located in the membranes of adjacent endothelial cells [185,186].

The understanding of drug delivery across the BBB should also consider the structure and selectivity of tight junction proteins. Claudin-5 from tight junctions (Figure 3) forms pores that mediate the paracellular permeability of molecules smaller than 800 Da [178]. Molecular modelling and simulation studies on the subject are extensively reviewed in [178]. Such an approach can lead to the identification of compounds that modulate properties of tight junctions such as specificity, pore size or permeability [178]. 

Collagen IV, a key component of BBB, can be degraded by MMP-9. During central nervous system inflammation, MMP expression, particularly MMP-9, is linked to BBB breakdown. Propofol (Table 2) is a sedative drug that reduces MMP-9 expression in human cerebral microvascular endothelial cells triggered by inflammatory factor TNF. Propofol can restore BBB integrity that has been harmed by TNF, and it also reduces TNF’s inhibitory action on collagen IV [179].

T2DM is a significant predictor of perioperative neurocognitive disorder. However, the mechanism of action is still understudied. Zhang et al. [180] investigated the treatment with TAK-242 (Table 2) on adult male db/db and db/m mice (a mouse model of type 2 diabetes mellitus) on tibial fracture surgery-induced hippocampal BBB damage. TAK-242 is a selective inhibitor of Toll-like receptor 4 (TLR4) [180]. This receptor promotes lipopolysaccharide-induced microglial activation and inflammatory cytokine levels in high glucose conditions. The study found that TLR4-mediated hippocampus inflammatory cytokine release, MMP/TIMP axis imbalance, and BBB rupture were improved by TLR4 inhibition [180].

Haemorrhagic transformation is a neurological disease that worsens as a result of an ischemic stroke. Although the chemical mechanism is unclear, studies show that Bradykinin 1 receptor (B1R) causes vascular toxicity. In a rat model of cerebral ischemia/reperfusion with type 1 diabetes, Sang et al. [187] investigated B1R expression in brain tissues [187]. According to the study findings, B1R-specific antagonists reduced haemorrhage volume and BBB disruption in diabetic patients in a dose-dependent manner, and the specific agonists increased it. Through ERK signalling, B1R was involved in ischemia-related bleeding and BBB degradation in diabetic rats. B1R-activated ERK1/2 stimulated NF-B activation, resulting in MMP-9 production and tight junction associated protein degradation. U0126 (1,4-Diamino-2,3-dicyano-1,4-bis(o-aminophenylmercapto)butadiene) and pyrrolidine dithiocarbamate (Table 2), both of which were tested, showed encouraging results. B1R-induced NF-B/p65 activation was decreased by U0126, an ERK inhibitor. In addition, following a stroke, this chemical restores BBB function. Pyrrolidine dithiocarbamate is an NF-B selective inhibitor that reduces the levels of MMP-9 mRNA and protein in haemorrhagic tissues [187].

APX3330 (Table 2) is a specific APE1/Ref-1 redox activity inhibitor that exhibits therapeutic benefits in T1DM stroke rats. In a study conducted by Yan et al. [166], rats with T1DM were given a temporary middle cerebral artery blockage; they were then treated with PBS or APX3330, and their BBB permeability was measured. According to the findings, APX3330 therapy for stroke in T1DM rats improved neurological functional outcome, BBB integrity, total vascular density, and other factors. Moreover, in cultured primary cortical neurons exposed to high glucose and oxygen-glucose deprivation, APX3330 therapy substantially reduced cell mortality and MMP-9 gene expression [166].

Soluble epoxide hydrolase (sEH), an enzyme that degrades epoxyeicosatrienoic acids (EETs), has various beneficial effects on vascular structure and function. According to Wu et al. [188] enhanced BBB vascular permeability was accompanied by overexpression of sEH and downregulation of 14,15-EET. The study concluded that decreased EET degradation caused by sEH inhibition might be a therapeutic strategy for slowing the course of BBB damage in diabetic mice through activation of the AMPK/HO-1 pathway [188].

The chemical structures of the previously reported compounds are shown in Table 2. Furthermore, we determined the BBB permeability of compounds. The logarithmic ratio of brain to plasma concentration of the drug (log BBB) was predicted by pkCSM, whereas the probability of the molecule passing through the BBB (BBB probability) was predicted by admetSAR 2.0 [189,190].

**Table 2:** Compound name, SMILES code, chemical 2D structure, pkCSM [189] (sourced from pkCSM-parmacokinetics server [191]) and admetSAR2.0 [190] (sourced from admetSAR web server [192]) predictions. Molecules with a logBB > 0.3 are believed to cross the BBB, whereas molecules with a logBB-1 are poorly dispersed to the brain, according to pkCSM. admetSAR2.0 estimates a probability, with 1 indicating that the molecules cross the BBB and 0 indicating that they do not.

Compound	pkCSM Numeric (log BBB)	admetSAR 2.0 BBB Probability	SMILES	Structure
propofol	0.497	+(0.99)	CC(C)C1=C(C(=CC=C1)C(C)C)O	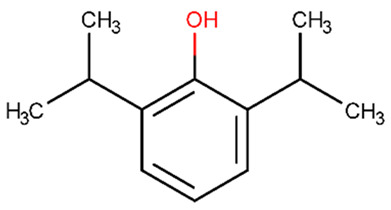
TAK-242	−0.715	+(0.97)	CCOC(=O)C1=CCCCC1S(=O)(=O)NC2=C(C=C(C=C2)F)Cl	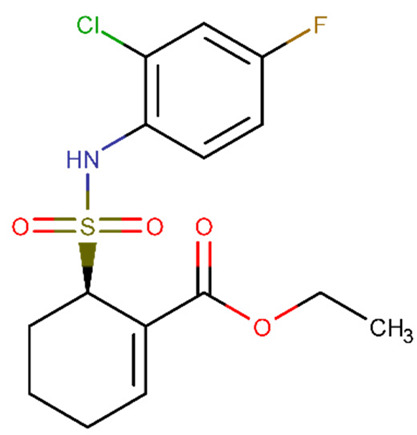
U0126	−0.967	+(0.97)	C1=CC=C(C(=C1)N)SC(=C(C#N)C(=C(N)SC2=CC=CC=C2N)C#N)N	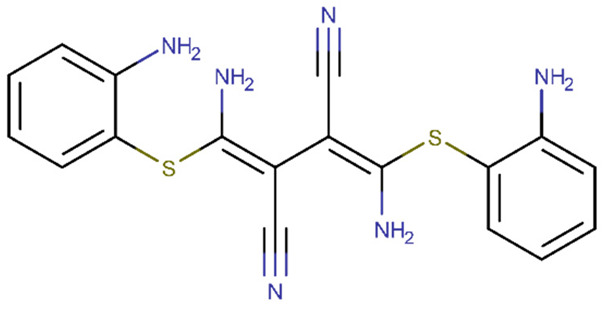
Pyrrolidine dithiocarbamate	0.041	+(0.98)	C1CCN(C1)C(=S)S	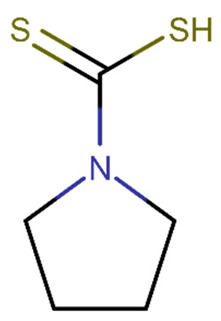
APX3330	−0.742	+(0.91)	CCCCCCCCCC(=CC1=C(C(=O)C(=C(C1=O)OC)OC)C)C(=O)O	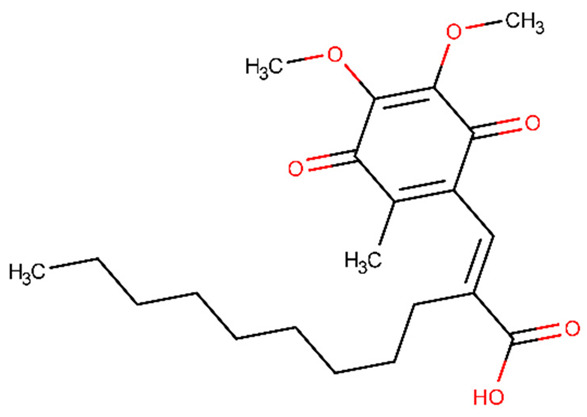

## 8. Natural Compounds That Prevent BBB Dysfunction in Diabetic Patients

Many neurological disorders have been found to be alleviated by natural compounds such as flavonoids, which modulate the signalling transduction cascades involved with BBB breakdown [193]. Understanding the actions of natural products through the molecular processes linked to BBB degradation in DM may pave the way for the discovery and development of new medicines to prevent the BBB breakdown.

In a quest for compounds able to preserve BBB integrity and proper function, Mamo et al. identified probucol as a promising compound [194]. The compound was effective in an insulin-resistant mice model obtained by administering a high fat, high fructose diet for 6 months. The mice presented a significantly increased BBB permeability relative to control due to the decrease in endothelial tight junction proteins (occludin-1 and zonula occludens (ZO)-1) expression. Probucol presented peripheral anti-inflammatory effects, reduced the levels of cholesterol supplied to BBB endothelial cells, and prevented the extravasation of IgG from blood to brain by restoring the levels of occluding-1 and ZO-1 [194]. 

Natural compounds can also present protective effects on the BBB under diabetic conditions; for example, resveratrol reduces BBB permeability [195] and berberine or patchouli alcohol reduce vascular damage [195]. Many other natural compounds that prevent BBB breakdown are summarized in [193]. The compounds belong to classes of alkaloids (berberine, caffeine), lipids (α-lipolic acid), phthalides (z-ligustilide), flavonoids (baicalin, genistein, pinocembrin, quercetin), phenols (caffeic acid phenethyl ester, curcumin, curculigoside A, forsythoside B, resveratrol, sesamol) or terpenes (6-O-acetyl shanzhiside methyl ester, astragaloside IV, ginkgolide B, ginsenoside Rb1, oleanolic acid, tanshinone IIA) and exhibit their BBB protective effects by modulating different transcription factors or signalling transduction cascades [193]. We should highlight that some of these compounds, such as curcumin, quercetin and berberine, also modulate molecular targets of diabetes, showing their multiple beneficial effects in DM. 

Kam et al. [193] also reviewed some structure–activity relationship (SAR) studies conducted on flavonoids [193]. For instance, the number of hydroxyl groups in the B-ring of 4-oxo-flavonoids correlates with their cytoprotective activity and their inhibitory activity on the expression of ICAM-1, an adhesion molecule involved in leukocyte recruitment [196]. 

Idebenone was used in combination with insulin to decrease BBB permeability in streptozotocin-induced diabetic rats [197]. The two molecules presented a synergistic effect in closing the tight junctions, upregulating the expression of occludin, claudin-5 and ZO-1, decreasing the levels of reactive oxygen species, and decreasing the levels of receptors for advanced end glycation products and for nuclear factor-kB [197].

Quercetin is a polyphenol that can limit high BBB permeability. Polyphenols decrease the adhesion of monocytes caused by hyperglycaemia to the level of brain endothelial cells [198]. Studies in animal models have shown how catechin metabolites are distributed in tissues, managing to cross the BBB [199]. This phenomenon was observed by measuring tissue permeability, thus showing the neuroprotective effect that can cause neurogenesis [200]. Quercitin resulted in a pK value (calculated using 1/Ki) of 7.62 ± 0.13 [201]. This natural compound is an inhibitor of GLUT-mediated glucose transport as well as a permeant ligand via GLUT [202]. Quercetin in the diet prevented diabetic mice from losing 59 percent and 63 percent of their GLUT 1 and GLUT 3 levels, respectively [169].

Prediction of isorutarine’s molecular properties showed that it cannot cross the BBB. However, its metabolites and various molecules resulting in its cleavage could cross the barrier and have a beneficial effect in the treatment of diabetes [117,203].

The natural compound kaempferol could present protective and preventive effects in diabetic complications [203,204]. Azevedo et al. showed that in the case of breast cancer cells, kaempferol inhibited glucose absorption via reducing GLUT1-mediated glucose uptake [205]. A study conducted by Martin et al. showed that kaempferol pK value calculated from absorbance was 7.30 ± 0.01, and a pK value calculated from 1/Ki was 7.51 ± 0.29 [201].

Curcumin decreased the transport activity of GLUT1 in a dose-dependent way by causing rapid reversible inhibition [206]. Curcumin may also help to prevent diabetes-related cerebral infarction, by inhibiting both the GLUT1 and GLUT3 transporters [207,208].

According to molecular docking research, rutin has a binding affinity (kcal/mol) of −13.101 on the GLUT1 transporter. This molecule forms a hydrogen bond with the backbone of GLUT1 through Thr137, Glu380, Asn415, Asn288 and Asn411 residues [209]. In human erythrocytes, rutin is a low-affinity inhibitor of glucose efflux via GLUT1 (Ki 0.1–0.3 mM) [202].

Berberine is a compound known to cross the BBB; this molecule modulates GLUT3 levels and has no effect on GLUT1 [169]. The activities involved in anti-inflammation and insulin resistance in the prefrontal cortex of diabetic rats were used to examine the influence of berberine on cognitive activity in diabetics. Berberine has the potential to boost GLUT3 expression and reverse the damage [210]. Even though some studies claim berberine has no impact on GLUT1, Cok’s research showed that berberine abruptly increases GLUT1 transport activity [211]. 

Catechins are also known to inhibit GLUT, especially GLUT1 [212,213]. 

In rats with type 1-like diabetes mellitus, Lee et al. found that ginseng extracts, gs-kg9 and gs-e3d, reduce BBB damage and thereby decrease apoptotic cell death of hippocampus neurons. The findings revealed that GS-KG9 and GS-E3D inhibited MMP-9 expression and activation, had dose-dependent antihyperglycemic action, and dramatically reduced BBB permeability and tight junction protein loss [214].

## 9. Databases and Web-Servers of Anti-Diabetic Compounds

Specific databases are a valuable tool in drug discovery, especially when it comes to in silico studies. We have identified three specific databases and web-servers of antidiabetic compounds. 

Anti-Diabetic Natural Compounds Database (ADNCD) collects and categorizes natural compounds according to their anti-diabetic modes of action (e.g., Akt phosphorylation, improving glucose uptake, insulin mimetic activity, insulin sensitizers), providing a single platform with advantages for diabetes researchers. The database also contains information on the physicochemical characteristics (miLogP**, absorption percent, Lipinski’s violation, etc.), and toxicity (mutagenic and tumorigenic risk, reproductive and irritant effect) of anti-diabetic natural compounds [215].

DiaNat-DB is a database containing 336 compounds from plant species that show anti-diabetic action in vitro or in vivo. This database provides the SMILES of the compound, the activity, plant family and genera, the use, and the country/region. That information can help future analysis, design, and development of novel anti-diabetic medicines. DiaNat-DB is freely available from this link: http://rdu.iquimica.unam.mx/handle/20.500.12214/1186, accessed on 10 October 2021 [216].

Dia-DB is an open web server that uses two techniques to predict the probability of a molecule to be utilized as an anti-diabetic drug. The web server compares the results with a selected library of anti-diabetic medications and experimental molecules based on similarity. Additionally, based on user-selected input compounds, Dia-DB conducts an inverse virtual screening against a set of proteins considered relevant targets for anti-diabetic components, such asperoxisome proliferator-activated receptor delta, aldose reductase, insulin receptor precursor, glucokinase, etc. The web server can be accessed at: https://bio-hpc.ucam.edu/dia-db/, accessed on 10 October 2021 [217].

## 10. Blood Brain Barrier Permeability Prediction Web Services

We identified a number of free web services that can estimate whether or not a chemical would pass the BBB. Prof. Xiang-Qun developed the BBB Predictor of COMPUTATIONAL CHEMICAL GENOMICS SCREENING CENTER website. Using the support vector machine (SVM), LiCABEDS algorithms and four types of fingerprints (MACCS, Openbabel (FP2), Molprint 2D, and Pubchem) of 1593 reported chemicals, this web-service will predict if a molecule can pass the BBB (BBB+) or cannot pass the BBB (BBB-). The chemical structures can be either loaded or drawn using the JSME molecular editor [218].

SwissADME, affiliated with the Swiss Institute of Bioinformatics, predicts ADME parameters, pharmacokinetics, drug-likeness, and medicinal chemistry compatibility of small molecules. Using 156 BBB permeable and 104 non-permeable compounds, this web service predicts a passive-BBB permeability model using a BOILED-Egg model. The parameters used for this model are WLOGP versus topological polar surface area. This model has a Matthews correlation coefficient of 0.79 [219,220].

pkCSM is a web service that employs a new method built on graph-based signatures to estimate the pharmacokinetic characteristics of compounds. These are used to train prediction algorithms by encoding distance patterns between atoms. The ratio of brain to plasma drug concentration is represented by the BBB permeability, which is given as logBBB. The method incorporates 320 chemicals, each of which has had its BBB determined experimentally [189].

admetSAR 2.0 is a free model for estimating and optimizing a small molecule’s chemical ADMET features. This web service estimates BBB permeability using a binary predictive algorithm that includes 1830 molecules in its training set. The model has an area under the receiver operating characteristic curve of 0.944, an accuracy of 0.907, a sensitivity of 0.921, and a specificity of 0.861 [190].

An example of pkCSM and admetSAR [189,190] usage is presented in Table 2 where we reported predicted BBB permeabilities of antidiabetic compounds discussed in Section 7. Additionally, we used the same tools to predict the BBB permeability of gymnemic acids presented in Section 3. The results in Table 3 show that all chemicals may pass the BBB, except for gymnemic acid VI.

**Table 3:** Predicted BBB permeability of gymnemic acids I-VII using pkCSM [189] (sourced from pkCSM-parmacokinetics web server [191]) and admetSAR2.0 [190] (sourced from admetSAR web server [192]). Their SMILES codes of the compounds are given as well.

**Compounds**	**pkCSM**	**admetSAR 2.0**	**SMILES**
gymnemic acid I,	−1.517	+0.843	CC=C(C)C(=O)OC1C(C2(C(CC1(C)C)C3=CCC4C5(CCC(C(C5CCC4(C3(CC2O)C)C)(C)CO)OC6C(C(C(C(O6)C(=O)O)O)O)O)C)COC(=O)C)O
gymnemic acid II,	−1.558	+0.91	CCC(C)C(=O)OC1C(C2(C(CC1(C)C)C3=CCC4C5(CCC(C(C5CCC4(C3(CC2O)C)C)(C)CO)OC6C(C(C(C(O6)C(=O)O)O)O)O)C)COC(=O)C)O
gymnemic acid III,	−1.652	+0.91	CCC(C)C(=O)OC1C(C2(C(CC1(C)C)C3=CCC4C5(CCC(C(C5CCC4(C3(CC2O)C)C)(C)CO)OC6C(C(C(C(O6)C(=O)O)O)O)O)C)CO)O
gymnemic IV,	−1.611	+0.84	CC=C(C)C(=O)OC1C(C2(C(CC1(C)C)C3=CCC4C5(CCC(C(C5CCC4(C3(CC2O)C)C)(C)CO)OC6C(C(C(C(O6)C(=O)O)O)O)O)C)CO)O
gymnemic acid, V,	−1.743	+0.84	CC=C(C)C(=O)OC1C(C2(C(CC1(C)C)C3=CCC4C5(CCC(C(C5CCC4(C3(CC2O)C)C)(C)CO)OC6C(C(C(C(O6)C(=O)O)O)O)O)C)CO)OC(=O)C(=CC)C
gymnemic VI,	−2.346	−0.78	CC=C(C)C(=O)OC1C(C2(C(CC1(C)C)C3=CCC4C5(CCC(C(C5CCC4(C3(CC2O)C)C)(C)CO)OC6C(C(C(C(O6)C(=O)O)O)OC7C(C(C(C(O7)CO)O)O)O)O)C)CO)O
gymnemic acid VII	−1.259	+0.84	CC1(CC2C3=CCC4C5(CCC(C(C5CCC4(C3(CC(C2(CC1O)CO)O)C)C)(C)CO)OC6C(C(C(C(O6)C(=O)O)O)O)O)C)C

B3Pred web server predicts and designs effective BBB penetrating peptides (B3PPs). The technique relies on 269 experimentally confirmed B3PPs from the B3Pdb database. This program uses the FASTA format of the peptides and estimates whether a peptide will penetrate the BBB. The results show a prediction score and some peptide features: hydrophobicity, hydropathicity, hydrophilicity, charge, and Mol wt [221]. 

LightBBB is a predictor of BBB permeability based on a collection of 7162 molecules with known BBB permeability. LightBBB uses the light gradient boosting machine technique and has an overall accuracy of 89 per cent. The model uses the SMILES code or an .SMI file, and the output indicates if a compound is or is not BBB permeable [222]. 

For a detailed perspective on the BBB permeability of candidate compounds, one could use MD simulations. Carpenter et al. performed reliable predictions of BBB permeability based on the potential of mean force calculations for candidate compounds through a lipid bilayer using MD simulations. The authors derived one-dimensional position-dependent diffusion coefficients based on MD trajectories. The effective permeability of a compound was determined based on its diffusion coefficient as corroborated with the free energy landscape. Their results were in good agreement with logBBB (blood-brain concentration ratio) and logPS (permeability surface-area product) of compounds [223]. A more recent study investigated the permeability of compounds by steered molecular dynamics simulations [224]. Using a simple lipid bilayer, Thai et al. [224] computed the non-equilibrium work required to pull a compound through the lipid membrane, leading to values in good correlation to logBBB or logPS. The method allows the usage of different membranes and brings insight on the energetic barriers and forces acting on the ligand when crossing the membrane [224].

## 11. Conclusions

T1DM and T2DM are metabolic disorders in which insulin secretion is reduced (T2DM) or no longer secreted due to pancreatic-cell death (T1DM). The diabetic condition alters BBB function and integrity, which further results in diabetes-related neurological complications. In the present review, we discussed research on natural and synthetic compounds that operate on therapeutic target proteins in DM and on the BBB. We approached three current research directions, namely the modulation of proteins involved in glucose metabolism or in insulin response, of proteins involved in the development and preservation of pancreatic β cells, and of proteins involved in BBB permeability preservation. The relevant druggable targets for each direction were identified, some of them being IR, SIRT6, AR, α-glucosidases, PPAR, SGLT, 11-HSD1, GFPT1, PTP1B, DPP-4, GKRP, GLUT2, glycerol-3-phosphate, GSK-3, PDK2, glucokinase, and HDAC. In the case of all targets, we presented bioinformatics studies aiming to determine natural and synthetic compounds that could regulate their function. Different methods were taken into account, namely QSAR, molecular docking and molecular dynamics. 

Only a few QSAR studies have investigated the effect of natural compounds on specific targets in diabetes such as α-glucosidase, GPR40, or AR receptors. According to the previously reported studies, andrographolide and flavone derivatives are active on the α-glucosidase target, while 3-aryl-3-ethoxypropanoic acid derivatives are GPR40 modulators.

The most popular protein targets approached by molecular docking studies are α-glucosidases and IR. Molecular docking studies predicted that natural compounds such as kaempferol, herbacetin, or herbacetin should present an enhanced affinity for receptors such as AR, IR, or SIRT6. Anthroquinonol and rutin presented good docking scores in interaction with the α-glucosidase receptor, while docosanol, tetracosanol, anthroquinonol and berberine presented good docking scores in interaction with α-amylase. 

Molecular dynamics studies showed that (i) shahidine, epicatechin, quercetin, isocolumbin, ellagic acid, and lutolin should inhibit α-amylase; (ii) curcumin and pipernonaline should form stable complexes with PPARγ; (iii) β-amyrine, teraxerol, 1-O-galloyl-β-D-glucose, corilagin, cosmosin, quercetin-3-galactoside and quercetin should modulate 11β-HSD1, GFPT1, PTP1B and SIRT6; (iv) shikonin could inhibit PTP1B; (v) apigenin and luteolin target HDAC1 and HDAC2; (vi) sophoraflavone G should inhibit SGLT1 and SGLT2. 

GLUT1, GLUT3, B1R, TLR4 or MMP-9 are relevant targets for preventing alterations of BBB permeability due to glycaemic variations in T1DM and T2DM patients. Numerous studies investigated compounds that could prevent BBB dysfunctions, some examples being propofol (synthetic compound) or berberine, genistein, quercetin, resveratrol or curcumin (natural compounds). The permeability of BBB can be modelled using different prediction servers such as SwissADME, pkCSM or admetSAR. 

The findings of reviewed simulation studies are encouraging, indicating that analysed compounds have potent inhibitory effects on specific receptors in DM treatment. These compounds might also be employed in in vitro and in vivo investigations or in future in silico studies as “lead-like” structures. Databases of natural compounds with anti-diabetic activity (ADNCD and DiaNat-DB) and Dia-DB webserver (predicts the possibility of a molecule used as an anti-diabetic drug) are helpful tools that may be used to find new anti-diabetic medicines or natural compounds. 

To summarize, natural substances may be a viable choice for T2DM management, as a therapy adjuvant, or to prevent diabetes-related dysfunctions; however, more studies are needed. Although insulin therapy is required for T1DM, natural or synthetic compounds such as propofol, APX3330, coumarin, quercetin, kaempferol, berberine, and others show promise in treating BBB damage caused by glucose swings, and therefore, should be investigated further.
